# Development and Validation of Machine Learning Models for Predicting Occult Nodal Metastasis in Early-Stage Oral Cavity Squamous Cell Carcinoma

**DOI:** 10.1001/jamanetworkopen.2022.7226

**Published:** 2022-04-13

**Authors:** Nathan Farrokhian, Andrew J. Holcomb, Erin Dimon, Omar Karadaghy, Christina Ward, Erin Whiteford, Claire Tolan, Elyse K. Hanly, Marisa R. Buchakjian, Brette Harding, Laura Dooley, Justin Shinn, C. Burton Wood, Sarah L. Rohde, Sobia Khaja, Anuraag Parikh, Mustafa G. Bulbul, Joseph Penn, Sara Goodwin, Andrés M. Bur

**Affiliations:** 1 Department of Otolaryngology–Head and Neck Surgery, University of Kansas Medical Center, Kansas City; 2 Department of Otolaryngology, Nebraska Methodist Health System, Omaha; 3 Department of Otolaryngology–Head and Neck Surgery, University of Iowa, Iowa City; 4 Department of Otolaryngology–Head and Neck Surgery, University of Missouri, Columbia; 5 Department of Otolaryngology–Head and Neck Surgery, Vanderbilt University, Nashville, Tennessee; 6 Department of Otolaryngology–Head and Neck Surgery, University of Minnesota, Minneapolis; 7 Department of Otolaryngology–Head and Neck Surgery, Massachusetts Eye and Ear Infirmary, Harvard University, Boston

## Abstract

**Importance:**

Given that early-stage oral cavity squamous cell carcinoma (OCSCC) has a high propensity for subclinical nodal metastasis, elective neck dissection has become standard practice for many patients with clinically negative nodes. Unfortunately, for most patients without regional metastasis, this risk-averse treatment paradigm results in unnecessary morbidity.

**Objectives:**

To develop and validate predictive models of occult nodal metastasis from clinicopathological variables that were available after surgical extirpation of the primary tumor and to compare predictive performance against depth of invasion (DOI), the currently accepted standard.

**Design, Setting, and Participants:**

This diagnostic modeling study collected clinicopathological variables retrospectively from 7 tertiary care academic medical centers across the US. Participants included adult patients with early-stage OCSCC without nodal involvement who underwent primary surgical extirpation with or without upfront elective neck dissection. These patients were initially evaluated between January 1, 2000, and December 31, 2019.

**Exposures:**

Largest tumor dimension, tumor thickness, DOI, margin status, lymphovascular invasion, perineural invasion, muscle invasion, submucosal invasion, dysplasia, histological grade, anatomical subsite, age, sex, smoking history, race and ethnicity, and body mass index (calculated as weight in kilograms divided by height in meters squared).

**Main Outcomes and Measures:**

Occult nodal metastasis identified either at the time of elective neck dissection or regional recurrence within 2 years of initial surgery.

**Results:**

Of the 634 included patients (mean [SD] age, 61.2 [13.6] years; 344 men [54.3%]), 114 (18.0%) had occult nodal metastasis. Patients with occult nodal metastasis had a higher frequency of lymphovascular invasion (26.3% vs 8.1%; *P* < .001), perineural invasion (40.4% vs 18.5%; *P* < .001), and margin involvement by invasive tumor (12.3% vs 6.3%; *P* = .046) compared with those without pathological lymph node metastasis. In addition, patients with vs those without occult nodal metastasis had a higher frequency of poorly differentiated primary tumor (20.2% vs 6.2%; *P* < .001) and greater DOI (7.0 vs 5.4 mm; *P* < .001). A predictive model that was built with XGBoost architecture outperformed the commonly used DOI threshold of 4 mm, achieving an area under the curve of 0.84 (95% CI, 0.80-0.88) vs 0.62 (95% CI, 0.57-0.67) with DOI. This model had a sensitivity of 91.7%, specificity of 72.6%, positive predictive value of 39.3%, and negative predictive value of 97.8%.

**Conclusions and Relevance:**

Results of this study showed that machine learning models that were developed from multi-institutional clinicopathological data have the potential to not only reduce the number of pathologically node-negative neck dissections but also accurately identify patients with early OCSCC who are at highest risk for nodal metastases.

## Key Points

**Question:**

Can clinicopathological variables be used to develop machine learning models that can predict occult nodal metastasis in patients with early-stage oral cavity squamous cell carcinoma (OCSCC)?

**Findings:**

In this diagnostic modeling study that included 634 patients with early OCSCC, machine learning models were developed that could predict occult nodal metastasis with a significantly higher degree of accuracy compared with tumor depth thresholds that are commonly used in clinical practice.

**Meaning:**

Findings of this study suggest that predictive models developed from clinicopathological variables have potential to ensure adequate treatment and reduce morbidity by correctly identifying patients with early OCSCC at highest risk for occult nodal metastasis.

## Teaser

This diagnostic study describes the development and validation of models that use clinicopathological data to predict occult nodal metastasis among patients with early oral cavity squamous cell carcinoma.

## Introduction

Regional metastasis in oral cavity squamous cell carcinoma (OCSCC) is associated with increased risk of recurrence and decreased survival.^1,2^ Neck dissection is indicated in patients with nodal disease that is evident at the time of diagnosis and in patients with advanced tumors.^3^ Treatment of patients with early OCSCC without clinical nodal disease is a subject of ongoing debate. Patients who have clinically node-negative disease but develop nodal progression have substantially worse outcomes than those who undergo upfront elective neck dissection (END).^4^ However, approximately 70% to 80% of patients who undergo END will have pathologically negative lymph nodes, potentially resulting in unnecessary surgical morbidity and increased health care costs for these patients.^5,6,7,8,9,10,11,12,13^

The decision to perform END in patients with cT1-2N0 OCSCC is commonly based on tumor thickness or depth of invasion (DOI), which has consistently demonstrated an association with risk of occult nodal metastasis.^4,14,15,16^ In light of the adverse implications of nodal recurrence for survival, however, any decision to observe the neck rather than perform neck dissection should be made in carefully selected, follow-up–adherent patients.^17^ Therefore, one of the primary goals of any predictive model in this setting is to optimize negative predictive value (NPV), with a focus on minimizing false-negative results and resultant observation of patients with occult lymph node metastasis. Use of machine learning presents an opportunity to improve patient selection and reduce the number of neck dissections performed in patients who have no pathological node (pN) metastasis without compromising oncological outcomes for patients who have occult nodal disease.

A previous study used data from the National Cancer Database to develop machine learning to predict occult nodal metastasis in early OCSCC.^18^ This model compares favorably to a model based on DOI, which is commonly used at many institutions. Herein, we expanded on this previous work by describing machine learning models that were based on retrospectively collected multi-institutional data from patients with early-stage oral cancer. Specifically, we aimed to develop and validate predictive models of occult nodal metastasis from clinicopathological variables that were available after surgical extirpation of the primary tumor and to compare predictive performance against DOI, the currently accepted standard. We hypothesized that these predictive models would outperform previously developed models by incorporating elements of primary tumor pathology that are not captured in the National Cancer Database.

## Methods

### Study Cohort

Data for this diagnostic modeling study were obtained from 7 institutions across the US (University of Kansas Medical Center, Kansas City; University of Missouri Health System, Columbia; University of Iowa Hospitals and Clinics, Iowa City; Vanderbilt University Medical Center, Nashville, Tennessee; Nebraska Methodist Health System, Omaha; University of Minnesota Medical Center, Minneapolis; and Massachusetts Eye and Ear Infirmary, Boston). All included patients were initially evaluated between January 1, 2000, and December 31, 2019. This study was approved by the institutional review board of all participating institutions, which waived the informed consent requirement because the study used retrospective, deidentified data. Data use agreements were established, if applicable, between the University of Kansas and the participating institutions. This study adheres to the Transparent Reporting of a Multivariable Prediction Model for Individual Prognosis or Diagnosis (TRIPOD) reporting guideline.^19^

Patients, aged 18 years or older, with early-stage (cT1-2) OCSCC without clinical evidence of nodal involvement (cN0) who were treated with primary surgery were identified. Patients were included if surgical extirpation of the primary tumor was performed and (1) END was performed to manage the regional lymph nodes, (2) the neck was observed with no evidence of regional recurrence after a minimum of 2 years of clinical follow-up, or (3) the neck was observed with regional recurrence occurring within 2 years of initial surgical therapy (eFigure 1 in the Supplement). Patients were excluded if they had a history of radiotherapy to the head and neck or previous neck dissection. Patients who were treated nonsurgically (eg, definitive radiotherapy) were also excluded, as were patients with regional or distant metastatic disease on preoperative physical examination or imaging.

### Outcome and Predictor Variables

The outcome of interest was pN metastasis. This outcome was defined as a clinically node-negative tumor that was identified in the lymph nodes after END or development of biopsy-confirmed regional metastatic disease within 2 years in patients whose necks were observed.

Variables of interest were clinical (age, sex, race and ethnicity, body mass index [calculated as weight in kilograms divided by height in meters squared], smoking history, and anatomic tumor site) and pathological (largest tumor dimension, DOI, muscle invasion, submucosal invasion, dysplasia, histological grade, involvement of margins, perineural invasion [PNI], and lymphovascular invasion [LVI]) variables. Race and ethnicity data were reported in the electronic health records of the participating institutions. The race and ethnic categories included Asian, Black, Hispanic, Native American, and White.

Pathologists at the participating institutions currently measure tumor DOI from the plumb line that is perpendicular to the horizon established by the basement membrane of the nearest normal squamous mucosa, which is consistent with the incorporation of DOI into the eighth edition of the American Joint Committee on Cancer staging manual.^20,21^ However, measurement of this variable was not standardized throughout the study period. Although DOI and thickness are not interchangeable, thickness is generally used preoperatively because DOI is assessed using the surgical specimen. However, both DOI and thickness are associated with lymph node metastases.^22^ For the purpose of this study, DOI and thickness were both collected, and DOI was used when available.

### Model Training and Statistical Analysis

A predictive model based on tumor DOI alone was developed such that tumors with depth at or greater than the depth threshold in millimeters were predicted to have occult nodal disease and tumors with depth less than the depth threshold were predicted to not have occult nodal disease. In this model, the depth threshold was used to recommend END. A threshold depth of 4 mm was chosen as the benchmark prediction against which the developed models were compared because this value has been shown to provide optimal NPV.^16^

Classification algorithms were developed to predict for pN positivity using variables that were available after the initial surgical resection. Models we evaluated included logistic regression, random forest, support vector machine classifier, and XGBoost. XGBoost was chosen over traditional gradient boosting algorithms because of the additional regularization protocol, which acts to control model complexity to prevent overfitting during the initial model training.

Before model training, dummy variables were generated for categorical features whose values were not directly related to one another. Variables with clear ordinality were label encoded to retain information of their relative position. Missing values for these features were assigned to the unknown category, which was assigned an integer value nearest to the mean.

The multi-institutional cohort was nonrandomly split into a model development cohort and an external validation cohort. Patients from the Nebraska Methodist Health System were designated as the external validation cohort to account for the potential nonrandom variation across the different institutes. The model development cohort was subsequently randomly split into 80% as the training set and 20% as the internal validation set. Samples were stratified such that the ratio of cases with pN metastasis was proportionally equal to the entire data set. Continuous features were standardized by removing the mean and scaling to unit variance. This standardization was done after the internal validation and training sets were completely isolated to prevent the internal validation set from retaining information about the distribution of the training set. To control for class imbalance, weights were applied to the minority class to ensure equal distribution of error during model development and to equalize the penalization for misclassification.

Hyperparameter optimization was done with repeated stratified K-fold cross-validation. The Tree Parzen Estimator method was used to identify hyperparameters that maximized the area under the receiver operating characteristic (ROC) curve.^23^ All models were developed in Python, version 3.7.10 using the sklearn,^24^ hyperopt,^25^ and XGBoost^26^ packages (Python Software Foundation).

The importance of each feature in the final prediction for ensemble models was determined by the number of times each feature was split. Coefficient magnitude was used as a proxy to compare the relative feature importance for models that were built with logistic regression and support vector machine classifier architectures. Discriminative ability of all developed models was initially evaluated on the internal validation set. Best-performing models of each algorithm were then applied to the external validation cohort. Overall performance of each model was assessed via the ROC area under the curve (AUC), which was calculated by the trapezoidal method. Associated CIs were generated, and pairwise comparison of the AUC between models was performed via the method described by DeLong et al.^27^ Additional evaluative criteria included NPV, positive predictive value, sensitivity, specificity, and accuracy. Number needed to screen was used to compare the marginal benefit of developed models that were associated with decreased misclassification.

Differences in patient characteristics that were measured on a continuous scale were compared using *t* tests, and categorical variables were evaluated using χ^2^ or Fisher exact tests. All statistical tests were 2-tailed, and the significance level for comparisons was set at *P* = .05. Data were analyzed between April 1, 2021, and November 1, 2021.

## Results

### Cohort Characteristics

We identified 911 patients with a diagnosis of early-stage (cT1-2) OCSCC who had clinically negative nodes (cN0) and had undergone primary surgical resection. Of these patients, 634 underwent END or observation of the neck, and a minimum of 2 years of clinical follow-up was available (Table 1). This group comprised 290 women (45.7%), 344 men (54.3%), and 589 White individuals (92.9%), with a mean (SD) age of 61.2 (13.6) years.

**Table 1.** Demographic and Clinical Differences in Pooled Cohort Between Patients With and Without Occult Nodal Metastasis

**Characteristic**	All, No. (%) (n = 634)	Occult nodes, No. (%)		*P* value
		Without (n = 520)	With (n = 114)	
Sex				
Female	290 (45.7)	241 (46.3)	49 (43.0)	.58
Male	344 (54.3)	279 (53.7)	65 (57.0)	
Age, mean (SD), y	61.2 (13.6)	60.9 (13.5)	62.7 (13.7)	.19
BMI, mean (SD)	27.7 (6.3)	28.0 (6.3)	26.7 (6.2)	.06
White race and ethnicity^a^	589 (92.9)	486 (93.5)	103 (90.4)	.22
Smoking				
Never smoker	233 (36.8)	185 (35.6)	48 (42.1)	.10
<10 pack-years	41 (6.5)	30 (5.8)	11 (9.6)	
≥10 pack-years	223 (35.2)	190 (36.5)	33 (28.9)	
LVI	72 (11.4)	42 (8.1)	30 (26.3)	<.001
PNI	142 (22.4)	96 (18.5)	46 (40.4)	<.001
Margins involved	47 (7.4)	33 (6.3)	14 (12.3)	.046
DOI, mean (SD), mm	5.7 (4.3)	5.4 (4.3)	7.0 (4.3)	<.001
Largest diameter, mean (SD), mm	15.9 (9.4)	15.5 (9.3)	17.4 (9.7)	.05
Grade				
I: Well differentiated	242 (38.2)	224 (43.1)	18 (15.8)	<.001
II: Moderately differentiated	327 (51.6)	256 (49.2)	71 (62.3)	
III: Poorly differentiated	55 (8.7)	32 (6.2)	23 (20.2)	
Subsite				
Tongue	449 (70.8)	358 (68.8)	91 (79.8)	.49
FOM	79 (12.5)	69 (13.3)	10 (8.8)	
Gum	29 (4.6)	26 (5.0)	3 (2.6)	
Buccal	24 (3.8)	20 (3.8)	4 (3.5)	
RMT	20 (3.2)	16 (3.1)	4 (3.5)	
Palate	14 (2.2)	13 (2.5)	1 (0.9)	
Lip	3 (0.5)	3 (0.6)	0 (0.0)	
Oral, NOS	16 (2.5)	15 (2.9)	1 (0.9)	

Abbreviations: BMI, body mass index (calculated as weight in kilograms divided by height in meters squared); DOI, depth of invasion; FOM, floor of mouth; LVI, lymphovascular invasion; NOS, not otherwise specified; PNI, perineural invasion; RMT, retromolar trigone.

^a^ Race and ethnicity data were reported in the electronic health records of the participating institutions. The race and ethnic categories included Asian, Black, Hispanic, Native American, and White. More than 90% of the patients were identified as White individuals.

A total of 114 patients (18.0%) had occult nodal disease. Of these patients, 94 (14.8%) had pN metastases (pN positive) that were identified on histological evaluation of neck dissection specimens. The remaining 20 patients (3.2%) had nodal relapse after neck observation. Univariate comparison of these 2 clinical groups revealed that patients with occult nodal metastasis had a higher frequency of LVI (26.3% vs 8.1%; *P* < .001), PNI (40.4% vs 18.5%; *P* < .001), and margin involvement by invasive tumor (12.3% vs 6.3%; *P* = .046) compared with those with pathologically negative nodes. In addition, patients with vs those without occult nodal metastasis had a higher frequency of poorly differentiated primary tumor (20.2% vs 6.2%; *P* < .001) and greater DOI (7.0 vs 5.4 mm; *P* < .001).

### Occult Nodal Phenotype

Given the significant differences seen on pairwise analysis, we sought to better understand the association of these variables with the clinical phenotype of patients with underlying occult nodal metastasis. To compare across variables of different units and magnitude, variables were standardized such that their means across the entire cohort were scaled to 0 and their SDs were scaled to 1. Variables were then ranked by their standardized mean difference between clinical phenotypes (eFigure 2 in the Supplement). Differences in LVI (SD difference, 0.54), histological grade (SD difference, 0.53), PNI (SD difference, 0.51), and DOI (SD difference, 0.32) appeared to contribute most to the occult nodal phenotype, when compared with the node-negative cohort.

### Predictive Performance of Machine-Learning Algorithms

Of the 634 total patients, data on 486 patients were gathered from the multi-institutional collaborative specifically for the purposes of this study, and this cohort was designated as the model development cohort. The data on the remaining 148 patients were collected by a single institution, and this cohort was designated as the external validation cohort. The model development cohort was randomly split into a training set and internal validation set. eTable 1 in the Supplement displays the demographic and clinical characteristics of these 3 groups.

**Figure 1.**
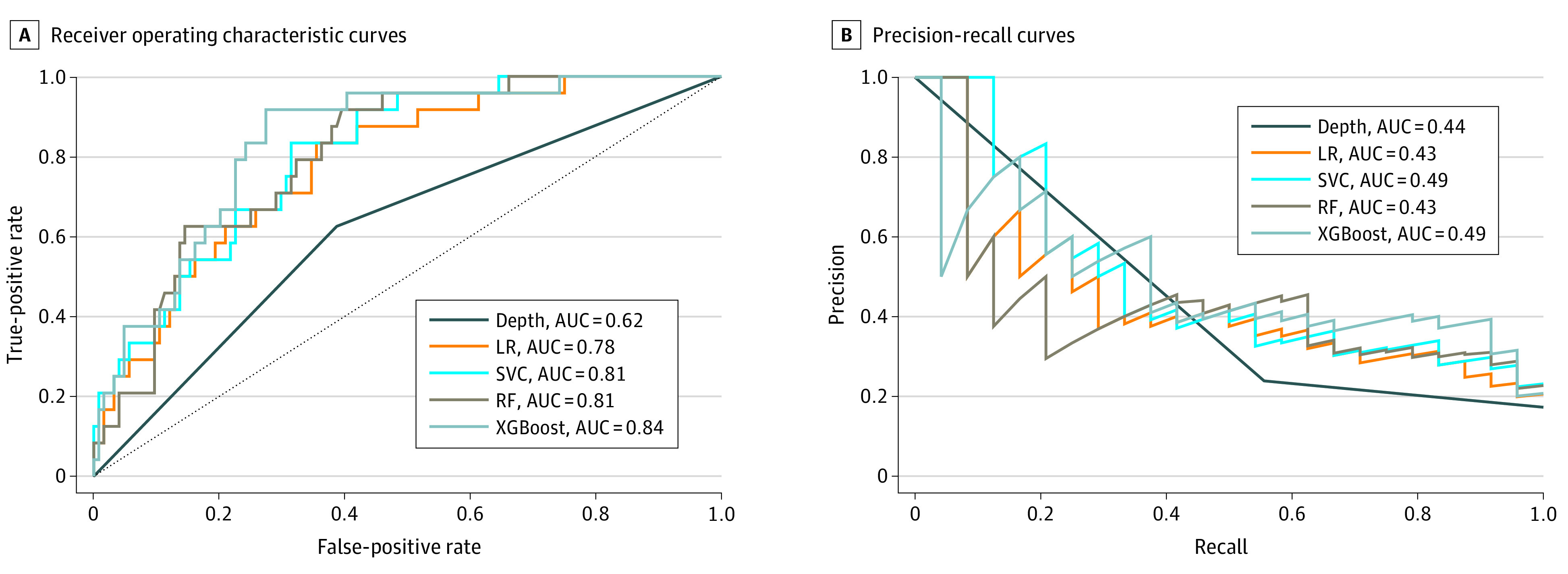
Predictive Ability of Depth of Invasion and the Machine Learning Models for Occult Nodal Metastasis in the External Validation Set A, Area under the receiver operating characteristic curves are shown for all 4 machine learning models (logistic regression [LR], random forest [RF], support vector machine classifier [SVC], and XGBoost) vs a tumor depth threshold. B, Precision recall curves for each of the models are shown with associated area under the curve (AUC).

Using patient characteristics gathered at the time of initial surgical resection, supervised machine learning models were trained to identify patients with occult nodal metastases. Figure 1 summarizes the predictive performance in the isolated external validation cohort of all 4 classification algorithms evaluated. A model generated from the XGBoost algorithm (ROC AUC = 0.84; 95% CI, 0.80-0.88) outperformed the tumor depth threshold (ROC AUC = 0.62; 95% CI, 0.57-0.67), logistic regression (ROC AUC = 0.78; 95% CI, 0.74-0.83), support vector machine classifier (ROC AUC = 0.81; 95% CI, 0.76-0.85), and random forest models (ROC AUC = 0.81; 95% CI, 0.76-0.85). All models outperformed the depth threshold for recommendation of END. Furthermore, these models outperformed DOI regardless of the depth threshold (eFigure 3 in the Supplement). Even a depth threshold that was specifically optimized for the external cohort, that of 3.2 mm, was only capable of achieving an ROC AUC of 0.716. Similarly, the XGBoost model had the best precision-recall performance with an AUC of 0.49 (95% CI, 0.43-0.53) (Figure 1B).

**Figure 2.**
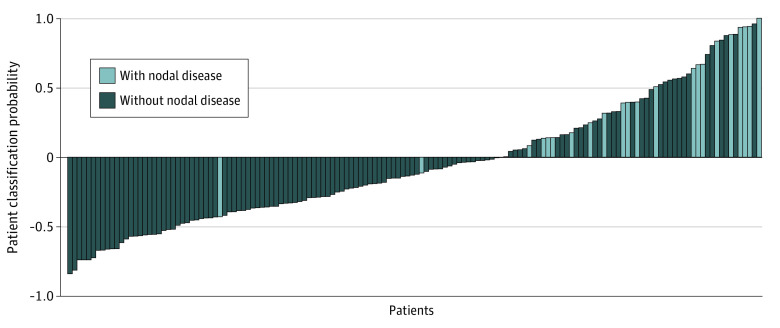
Relative Distance of Each Patient in the External Validation Cohort From the Decision Threshold for the Developed XGBoost Model The decision threshold was normalized to 0. Probability values were normalized as a relative distance from the largest absolute probability and the decision boundary.

Decision thresholds for each model were optimized using the Youden index (Table 2). At this optimized threshold, the XGBoost model had a sensitivity of 91.7%, a specificity of 72.6%, a positive predictive value of 39.3%, and an NPV of 97.8%. This resulted in misclassification of only 8.3% of patients with pN-positive disease compared with 37.5% of patients with the DOI threshold. In addition, the XGBoost model misclassified only 27.4% of patients with pN-negative disease compared with 38.7% of patients with the DOI threshold. As a result of this decreased misclassification, the number needed to screen to correctly identify additional patients with pN-positive disease would be 21.0, and the number needed to screen to correctly identify additional patients with pN-negative disease, thereby avoiding END, would be 10.6. Figure 2 displays the relative distance of each patient from the decision threshold for the XGBoost model as identified by their classification probability. Relative distance from the decision threshold for the remaining models in the external validation cohort are shown in eFigure 4 in the Supplement. Performance of all models in the model development cohort is shown in eTable 2 and eFigure 5 in the Supplement.

**Table 2.** Performance of Each Predictive Model on the External Validation Cohort^a^

	Depth	LR model	SVC model	RF model	XGBoost model
ROC AUC	0.619	0.783	0.806	0.805	0.838
*P* value	NA	<.001	<.001	<.001	<.001
Sensitivity, %	62.5	83.3	83.3	91.7	91.7
Specificity, %	61.3	64.5	68.6	60.5	72.6
PPV, %	23.8	31.3	33.9	31.0	39.3
NPV, %	89.4	95.2	95.5	97.4	97.8
Accuracy, %	61.5	67.6	71.0	65.5	75.7
Misclassified pN positive, %	37.5	16.7	16.7	8.3	8.3
NNS to identify pN positive, %	NA	29.6	29.6	21.0	21.0
Misclassified pN negative, %	38.7	35.5	31.5	39.5	27.4
NNS to avoid END	NA	37.0	16.5	NA	10.6

Abbreviations: AUC, area under curve; END, elective neck dissection; LR, logistic regression; NA, not applicable; NNS, number needed to screen; NPV, negative predictive value; pN, pathological node; PPV, positive predictive value; RF, random forest; ROC, receiver operating characteristic; SVC, support vector machine classifier.

^a^ Decision thresholds were optimized using the Youden index.

### Variable Importance

**Figure 3.**
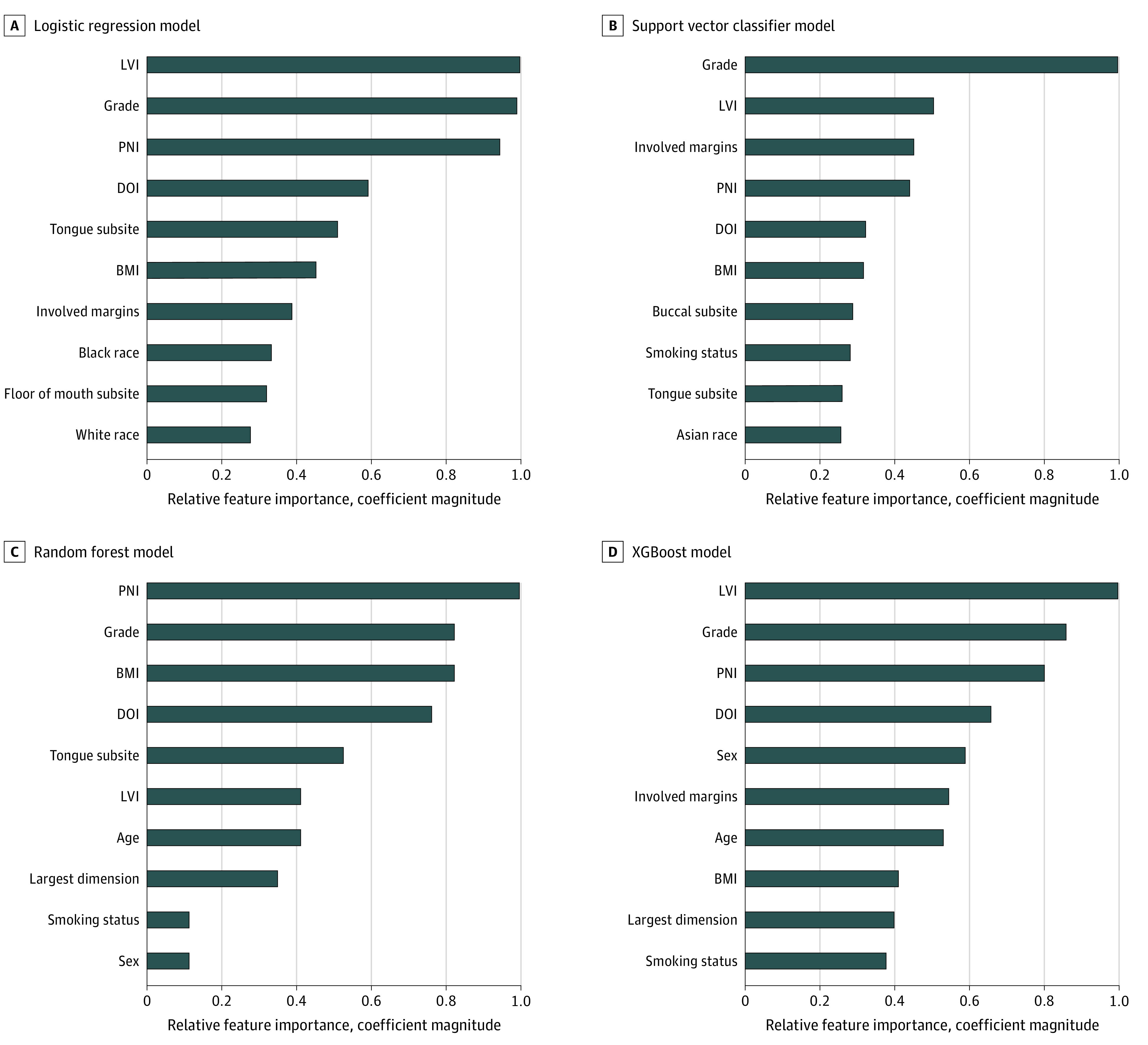
Relative Feature Importance of the Top 10 Features for Each Model Developed For the ensemble models, importance was determined by the number of times each feature was split. For the logistic regression and support vector machine classifier models, coefficient magnitude was used as a proxy for feature importance. Feature importance values were normalized against the highest performing feature for each model. BMI indicates body mass index (calculated as weight in kilograms divided by height in meters squared); DOI, depth of invasion; LVI, lymphovascular invasion; and PNI, perineural invasion.

Figure 3 shows the variable importance for all 4 of the developed models. For all models, histological grade was ranked in the top 2 most important features. Similar to the findings from the phenotypic analysis of the pooled cohorts, LVI, PNI, and DOI were consistently ranked among the most important features for each of the models.

## Discussion

Given the known survival advantage associated with END vs observation with salvage therapeutic neck dissection for patients with early-stage OCSCC, the ability to decrease pathologically node-negative neck dissections is contingent on the accurate identification of patients with occult nodal disease.^4^ This is especially true in the context of strong evidence that salvage therapeutic neck dissection is not oncologically equivalent to END, which furthers the support for aggressive upfront treatment of the neck. However, most patients who undergo neck dissection may have no pN disease, which results in no tangible benefit for individual patients and carries potential morbidity, including spinal accessory nerve dysfunction, hematoma, infection, and an unsightly neck scar.^9^

To ameliorate this dissonance, tumor thickness and DOI have been used to better identify patients who would benefit most from END. Numerous decision thresholds have been examined, and a depth threshold of 4 mm offers optimal NPV and is most used by surgeons.^16^ Although it is an improvement over more risk-averse treatment paradigms that offer END to all patients, its discriminative performance is underwhelming.

In this diagnostic predictive modeling study, we found that machine learning models that were developed from multi-institutional data outperformed DOI, the commonly used screening tool. Using a range of clinicopathological variables, we found that these models had the potential to not only decrease the number of patients who undergo pathologically node-negative neck dissection but also to better identify those at highest risk for occult nodal disease. Specifically, when compared against the DOI threshold, the XGBoost machine learning model correctly spared 1 additional patient for every 10.6 patients screened from undergoing END. Moreover, for every 21.0 patients screened, this model would correctly identify 1 additional patient with occult nodal metastasis compared with DOI.

In the initial pooled cohort analysis, we found that the occult nodal phenotype was largely defined by differences in LVI, histopathological grade, PNI, and DOI. In concordance, these were the 4 most important features of the best performing XGBoost model. Specifically, of all of the variables, LVI was shown to have the strongest discriminatory potential. This tumor characteristic identified on histological evaluation has been shown to be associated with higher rates of locoregional recurrence in patients with OCSCC.^28^ At the individual cell level, movement beyond an endothelial-lined boundary is associated with with epithelial-to-mesenchymal transition.^29^ In squamous cell carcinoma of the head and neck, a partial epithelial-to-mesenchymal transition expression signature is predictive of nodal metastases, LVI, and extranodal extension.^30,31^ This sequential pathway of epithelial-to-mesenchymal transition, LVI, and subsequent regional lymph node deposition is mechanistically sound because endothelial system invasion would be critical for greater than local travel and subsequent regional lymph node dissemination. Although conceptually similar, PNI and histological differentiation have both been shown to be independently associated with a higher rate of lymph node metastasis.^32,33,34,35,36,37,38,39^ As such, the best performing model was able to incorporate these previously identified associations to generate well-informed predictions of regional metastasis at the individual patient level.

With growing evidence to support the use of omic data to predict metastases in other cancers, merging extrinsic phenotypic characteristics, such as those used in this study, with upstream intrinsic genotypic biomarkers would likely augment this already strong performance.^40,41,42,43,44,45^ For example, the 2 patients who were misclassified in the external validation cohort had clinicopathologic features that were suggestive of low risk for nodal metastasis. For these patients, their genotypic profile could reveal a propensity for an aggressive phenotype that had yet to manifest clinically. As such, integrating these omic features in model development could result in more robust representations of individual patients and would likely allow for improved risk estimation across a greater range of clinical end points. Looking toward the future, the continued development and refinement of models that use artificial intelligence methodologies have the potential to substantially improve clinical decision-making in this and other areas of head and neck surgery.

### Limitations

This study has 2 limitations. The first limitation relates to the pathologic assessment of nodal disease. Routine histopathological evaluation involves bivalving grossly identified lymph nodes and staining with hematoxylin and eosin.^46^ This process may not capture micrometastatic disease that has been found to be present using more sophisticated techniques of specimen analysis.^47^ Clearance of this micrometastatic disease may account for a survival benefit that has been reported in patients who underwent END, even when no pN disease was identified.^48,49^ In this sense, pN positivity and nodal recurrence may not truly be equivalent, and it is impossible to retrospectively ascertain whether the 72.6% of patients who may have been spared from neck dissection by the machine learning model in our study would have had durable regional control with observation of the neck. However, many of the studies that lent support to the use of DOI to guide management of the neck similarly used pN status as an acceptable primary outcome, and routine pathological examination is considered to be acceptable in clinical practice to guide adjuvant therapy and prognostication.^14,15^ Thus, we believe that these 2 outcomes are sufficiently similar for the purposes of this study.

The second limitation is the lack of clarity regarding DOI. This factor differs from tumor thickness in its method of measurement and its predictive ability.^21^ Although current synoptic reporting has standardized the calculation and reporting of DOI, this was not consistently true at the participating institutions until after the publication of the eighth edition of the American Joint Committee on Cancer staging manual, which incorporated DOI into OCSCC staging. Pathological rereview may shed light on previously performed surgeries; however, this is costly and may not reflect clinical data.

## Conclusions

Accurate prediction of occult nodal metastasis in early oral cancer may potentially spare patients from morbidity associated with questionably beneficial neck dissection and ensure adequate treatment for occult nodal disease. In this study, we developed predictive models with machine learning methodologies that incorporated a wide range of clinicopathological features. These models predicted nodal metastasis with greater accuracy than a model based on DOI alone, which is the current criterion standard. This DOI threshold was initially developed to reduce pathologically node-negative ENDs, but the machine learning models we developed were not only capable of reducing this number but also far superior at correctly identifying patients at the highest risk for occult nodal disease.

## Notes

Supplement. **eTable 1.** Characteristics of Training, Internal Validation, and External Validation Cohort **eTable 2.** Performance of Developed Models on Training and Internal Validation Cohort **eFigure 1.** Inclusion Criteria and Categorization of Patients **eFigure 2.** Comparison of Variables That Define Occult Nodal Phenotype in Pooled Cohort **eFigure 3.** Discriminative Ability of Binary Tumor Depth Thresholds Across Range of Possible Values **eFigure 4.** Relative Probabilities in Relation to Decision Boundaries in External Validation Cohort for Logistic Regression, Support Vector Classifier, and Random Forest Models **eFigure 5.** Model Development Cohort Relative Probabilities in Relation to Decision Boundaries for Logistic Regression, Support Vector Classifier, Random Forest, and XGBoost Models
