# Supplementary material for: Development and Validation of Machine Learning Models for Predicting Occult Nodal Metastasis in Early-Stage Oral Cavity Squamous Cell Carcinoma
Source: JAMA Netw Open. 2022 Apr 13;5(4):e227226. doi: 10.1001/jamanetworkopen.2022.7226 (PMC9008495; doi:10.1001/jamanetworkopen.2022.7226)
Supplement: Supplement. — eTable 1. Characteristics of Training, Internal Validation, and External Validation Cohort eTable 2. Performance of Developed Models on Training and Internal Validation Cohort eFigure 1. Inclusion Criteria and Categorization of Patients eFigure 2. Comparison of Variables That Define Occult Nodal Phenotype in Pooled Cohort eFigure 3. Discriminative Ability of Binary Tumor Depth Thresholds Across Range of Possible Values eFigure 4. Relative Probabilities in Relation to Decision Boundaries in External Validation Cohort for Logistic Regression, Support Vector Classifier, and Random Forest Models eFigure 5. Model Development Cohort Relative Probabilities in Relation to Decision Boundaries for Logistic Regression, Support Vector Classifier, Random Forest, and XGBoost Models [file jamanetwopen-e227226-s001.pdf]

Supplementary Online Content

Farrokhian N, Holcomb AJ, Dimon E, et al. Development and validation of machine learning models for predicting occult nodal metastasis in early-stage oral cavity squamous cell carcinoma. *JAMA Netw Open*. 2022;5(4):e227226. doi:10.1001/jamanetworkopen.2022.7226

eTable 1. Characteristics of Training, Internal Validation, and External Validation Cohort

eTable 2. Performance of Developed Models on Training and Internal Validation Cohort

eFigure 1. Inclusion Criteria and Categorization of Patients

eFigure 2. Comparison of Variables That Define Occult Nodal Phenotype in Pooled Cohort

eFigure 3. Discriminative Ability of Binary Tumor Depth Thresholds Across Range of Possible Values

eFigure 4. Relative Probabilities in Relation to Decision Boundaries in External Validation Cohort for Logistic Regression, Support Vector Classifier, and Random Forest Models

eFigure 5. Model Development Cohort Relative Probabilities in Relation to Decision Boundaries for Logistic Regression, Support Vector Classifier, Random Forest, and XGBoost Models

This supplementary material has been provided by the authors to give readers additional information about their work.

© 2022 Farrokhian N et al. *JAMA Network Open.*

eTable 1. Characteristics of Training, Internal Validation, and External Validation Cohort

	Training (n = 388)	Internal Validation (n = 98)	External Validation (n=148)
Sex			
Female	179 (46.1)	42 (42.9)	69 (46.6)
Male	209 (53.9)	56 (57.1)	79 (53.4)
Age, y	60.7 (13.4)	59.2 (13.7)	63.8 (13.7)
BMI, kg/m^2	27.1 (5.8)	27.3 (5.7)	29.7 (7.5)
Race			
White	359 (92.5)	89 (90.8)	141 (95.3)
Other	29 (7.5)	9 (9.2)	7 (4.7)
Smoking			
Never smoker	138 (35.6)	39 (39.8)	56 (37.8)
<10 pack years	28 (7.2)	8 (8.2)	5 (3.4)
>10 pack years	148 (38.1)	36 (36.7)	39 (26.4)
Occult Nodes	72 (18.6)	18 (18.4)	24 (16.2)
LVI	49 (12.6)	11 (11.2)	12 (8.1)
PNI	96 (24.7)	25 (25.5)	21 (14.2)
Margins involved	33 (8.5)	8 (8.2)	6 (4.1)
DOI, mm	6.0 (4.6)	6.2 (4.4)	4.8 (3.6)
Largest diameter, mm	16.3 (9.2)	16.3 (9.4)	14.7 (9.7)
Grade			
I: Well differentiated	145 (37.4)	42 (42.9)	55 (37.2)
II: Moderately differentiated	205 (52.8)	47 (48.0)	75 (50.7)
III: Poorly differentiated	30 (7.7)	9 (9.2)	16 (10.8)
Subsite			
Tongue	280 (72.2)	77 (78.6)	92 (62.2)
FOM	42 (10.8)	12 (12.2)	25 (16.9)
Gum	14 (3.6)	0 (0.0)	15 (10.1)
Buccal	17 (4.4)	0 (0.0)	7 (4.7)
RMT	12 (3.1)	4 (4.1)	4 (2.7)
Palate	7 (1.8)	2 (2.0)	5 (3.4)
Lip	3 (0.8)	0 (0.0)	0 (0.0)
Oral, NOS	13 (3.4)	3 (3.1)	0 (0.0)

Abbreviations: pN+, pathologically node positive; LVI, lymphovascular invasion; PNI, perineural invasion; DOI, depth of invasion; FOM, floor of mouth; RMT, retromolar trigone; BMI, body mass index.

© 2022 Farrokhian N et al. *JAMA Network Open.*

eTable 2. Performance of Developed Models on Training and Internal Validation Cohort

		Depth	LR	SVC	RF	XGB
Training	ROC AUC	0.579	0.749	0.765	0.737	0.877
	p-value	-	<0.001	<0.001	<0.001	<0.001
	Sensitivity (%)	66.7	88.9	83.3	80.6	83.3
	Specificity (%)	49.1	56.0	63.9	64.9	77.9
	PPV (%)	23.0	31.5	34.5	34.3	46.2
	NPV (%)	86.6	95.7	94.4	93.6	95.4
	Accuracy (%)	52.3	62.1	67.5	67.8	78.9
Internal Validation	ROC AUC	0.633	0.790	0.785	0.764	0.899
	p-value	-	<0.001	<0.001	<0.001	<0.001
	Sensitivity (%)	77.8	88.9	66.7	77.8	94.4
	Specificity (%)	48.8	67.5	82.5	77.5	77.5
	PPV (%)	25.5	38.1	46.2	43.8	48.6
	NPV (%)	90.7	96.4	91.7	93.9	98.4
	Accuracy (%)	54.1	71.4	79.6	77.6	80.6

Decision thresholds were optimized using the Youden index. Abbreviations: ROC, receiver operator characteristic; AUC, area under curve; PPV, positive predictive value; NPV, negative predictive value.

© 2022 Farrokhian N et al. *JAMA Network Open.*

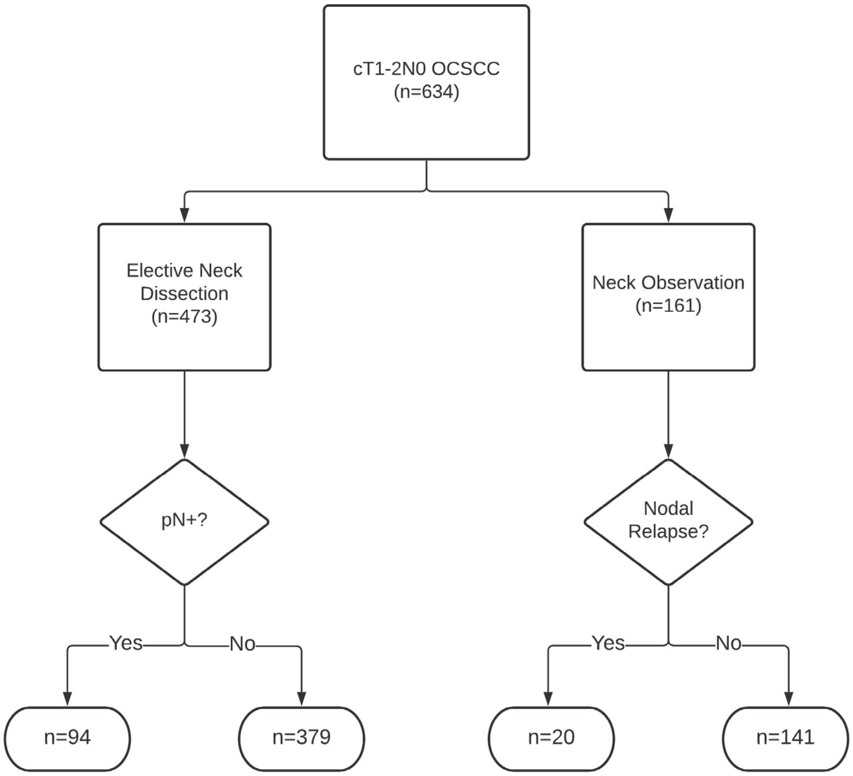

eFigure 1. Inclusion Criteria and Categorization of Patients

For patients whose necks were observed, nodal relapse was defined as regional recurrence within two years of initial surgical ablation. Negative nodal relapse was defined as no regional recurrence within the first two years after ablation. Those without nodal relapse and less than two years follow-up were excluded from the analysis.

© 2022 Farrokhian N et al. *JAMA Network Open.*

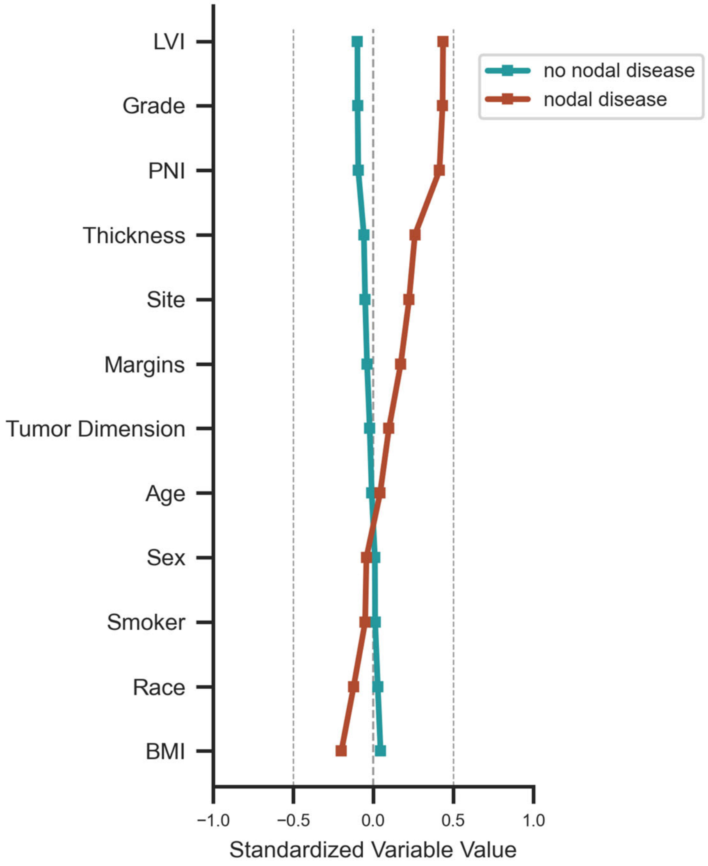

eFigure 2. Comparison of Variables That Define Occult Nodal Phenotype in Pooled Cohort

Variables were standardized by removing the mean and scaling to unit variance. In other words, means across the pooled cohort were set to zero and the standardized variable value represents the standard deviation from this mean for each phenotype. Margins signifies margin involvement by invasive tumor at time of initial surgical resection. Abbreviations: LVI, lymphovascular invasion; PNI, perineural invasion; BMI, body mass index.

© 2022 Farrokhian N et al. *JAMA Network Open.*

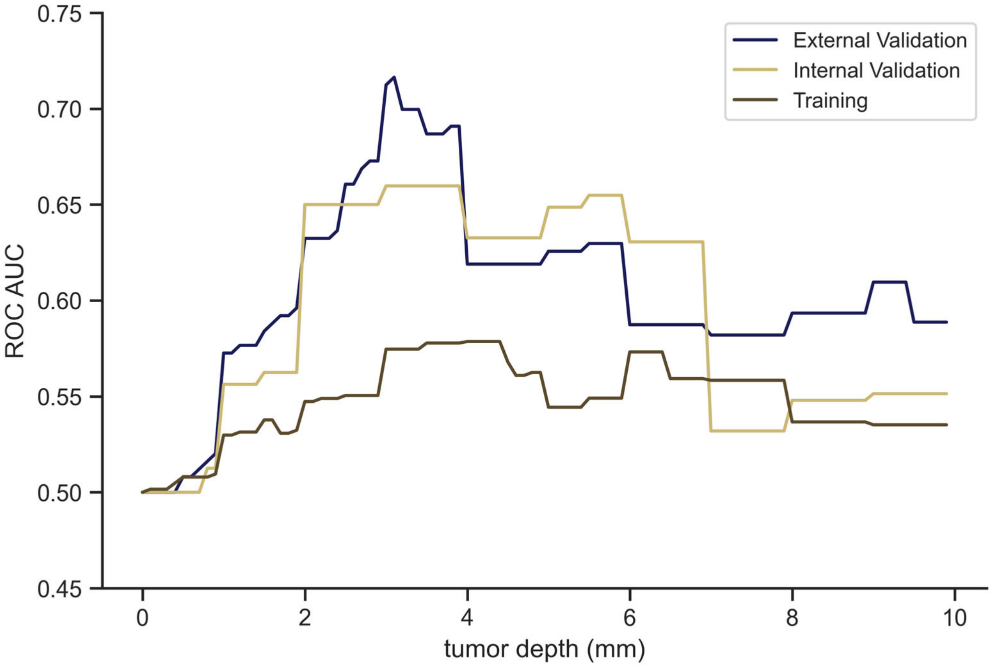

eFigure 3. Discriminative Ability of Binary Tumor Depth Thresholds Across Range of Possible Values

Range of discriminative ability of binary tumor depth thresholds to identify occult nodal metastasis as measured by area under the receiver operator characteristic curve for the training, internal validation, and external validation cohorts.

© 2022 Farrokhian N et al. *JAMA Network Open.*

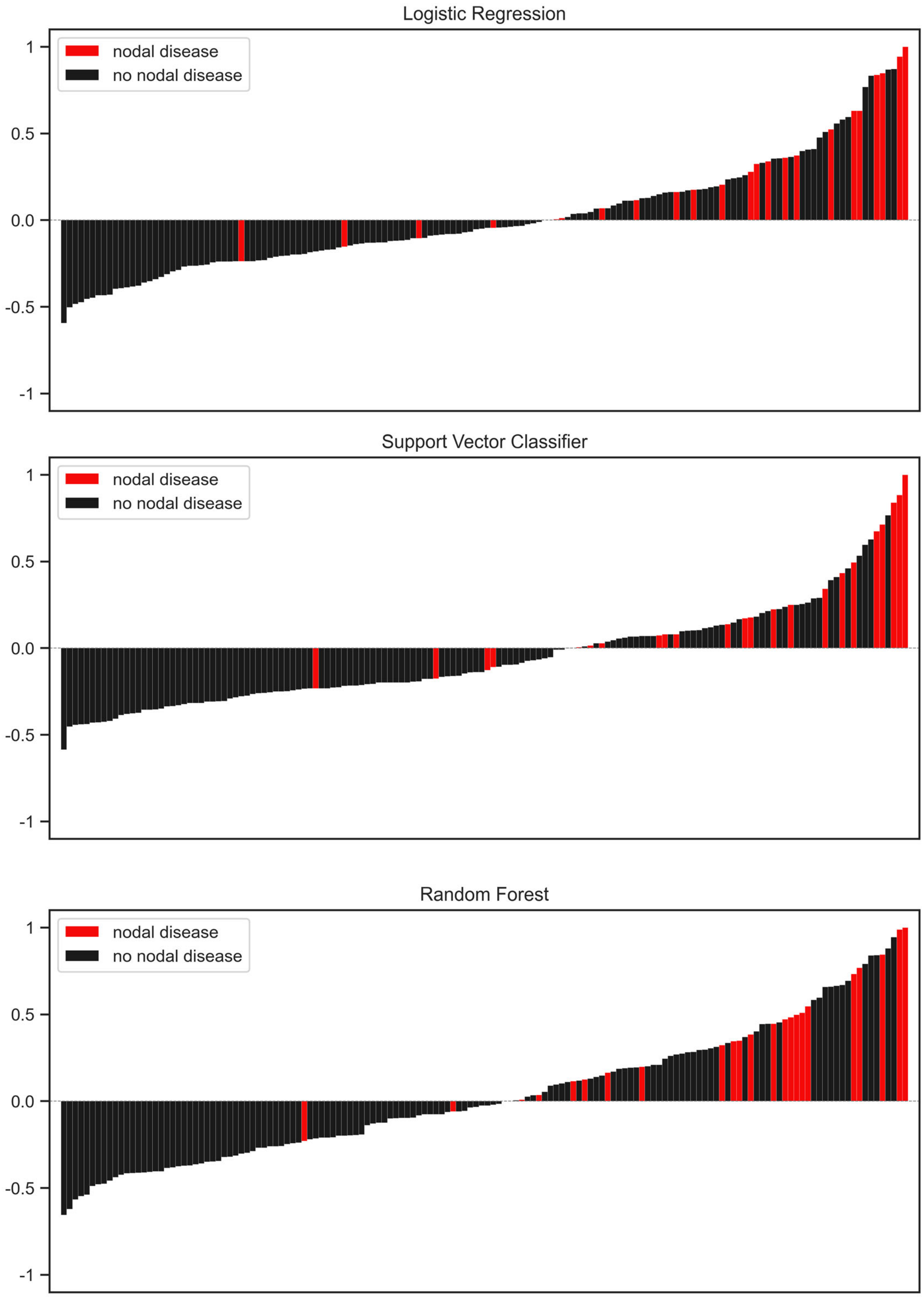

eFigure 4. Relative Probabilities in Relation to Decision Boundaries in External Validation Cohort for Logistic Regression, Support Vector Classifier, and Random Forest Models

Decision threshold was normalized to zero. Largest absolute probability was scaled to one and all other values were normalized as their relative distance between this value and zero.

© 2022 Farrokhian N et al. *JAMA Network Open.*

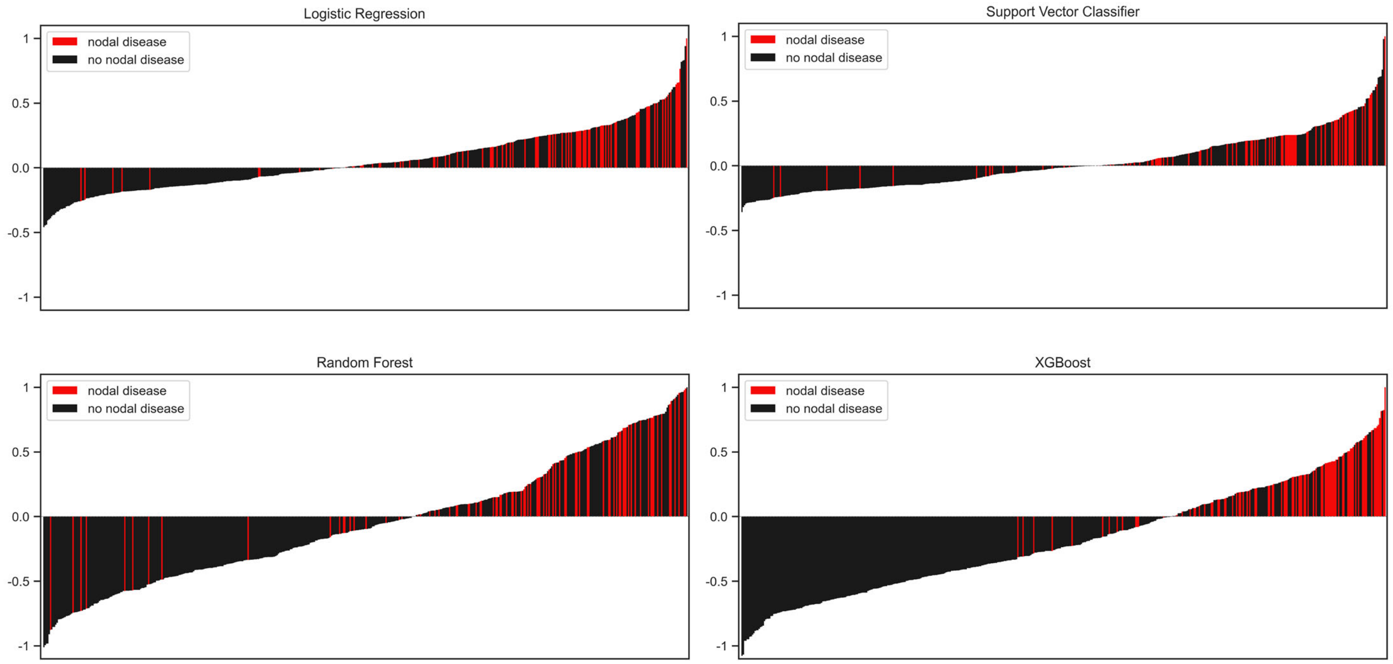

eFigure 5. Model Development Cohort Relative Probabilities in Relation to Decision Boundaries for Logistic Regression, Support Vector Classifier, Random Forest, and XGBoost Models

Decision threshold was normalized to zero. Largest absolute probability was scaled to one and all other values were normalized as their relative distance between this value and zero.

© 2022 Farrokhian N et al. *JAMA Network Open.*
